# Clinico‑pathological profile of oral squamous cell carcinoma: a five‑year analysis of 150 cases at Alexandria University

**DOI:** 10.1186/s12903-026-09057-0

**Published:** 2026-07-04

**Authors:** Abdalla Zaitoun, Mariam A. R. A. Mohamed, Tamer S. Hassan, Ahmed Saied Mohamed Mahmoud Marie, Mohamed Abdeldayem

**Affiliations:** Cranio-Maxillofacial and Plastic Surgery, Faculty of Dentistry, Alexandria University, Alexandria, Egypt

**Keywords:** Oral squamous cell carcinoma, AJCC staging, Depth of invasion, Extranodal extension, Histopathological risk factors, Survival outcomes

## Abstract

**Background:**

Oral Squamous Cell Carcinoma exhibits variable clinicopathological behavior and prognosis across different geographic populations. Limited data are available regarding the clinicopathological characteristics and prognostic factors of this disease in Middle Eastern populations, particularly in Egypt, where late presentation and advanced disease stages remain common challenges.

**Aim of study:**

To evaluate the clinicopathological characteristics of Oral squamous cell carcinoma and assess the prognostic significance of American Joint Committee on Cancer 8th edition staging, histopathological parameters, and combined risk patterns on survival and recurrence in an Egyptian cohort.

**Methods:**

This retrospective study included 150 Oral squamous cell carcinoma patients treated at Alexandria University between 2018 and 2022. Tumors were re-staged according to American Joint Committee on Cancer 8th edition criteria. Clinical, histopathological, and follow-up data were analyzed, including tumor differentiation, surgical margin status, perineural invasion, lymphovascular invasion, depth of invasion, and extranodal extension. Recurrence and survival outcomes were correlated with clinicopathological and combined histopathological risk factors.

**Results:**

Mean age of patients was approximately 56 years, with tongue being the commonest tumor site. Re-staging according to American Joint Committee on Cancer 8th edition resulted in significant stage migration toward advanced disease stages, particularly stage IVb. Moderately differentiated tumors represented the commonest histopathological grade. Advanced AJCC stages, depth of invasion > 10 mm, positive surgical margins, higher histological grades, positive extranodal extension, and perineural and lymphovascular positivity were significantly associated with poorer survival and higher recurrence rates. Smoking and positive family history were also significantly associated with adverse outcomes. In contrast, traditional TNM staging showed no significant association with recurrence or survival outcomes.

**Conclusion:**

American Joint Committee on Cancer 8th edition staging demonstrated improved risk stratification and stronger associations with recurrence and survival outcomes compared to traditional TNM staging in Oral Squamous Cell Carcinoma patients. Depth of invasion, extranodal extension, and the combined histopathological risk pattern were strongly associated with aggressive tumor behavior and poorer outcomes. These findings provide valuable data from an Egyptian and Middle Eastern population and support the integration of updated staging systems with histopathological risk assessment in Oral Squamous Cell Carcinoma management.

## Introduction

Oral cancer (OC) is a malignant neoplastic process affecting the oral cavity and oropharyngeal region. It is a global public health issue as it is the eighth most common cancer (> 300,000 cases annually) [1], associated with high mortality and significant functional and aesthetic morbidity [2]. The incidence and mortality rates of OC vary globally and are higher in developing nations, as India and other South/eastern Asia regions, France, Slovenia, Slovakia and Hungary [3–5].

**Fig. 1:**
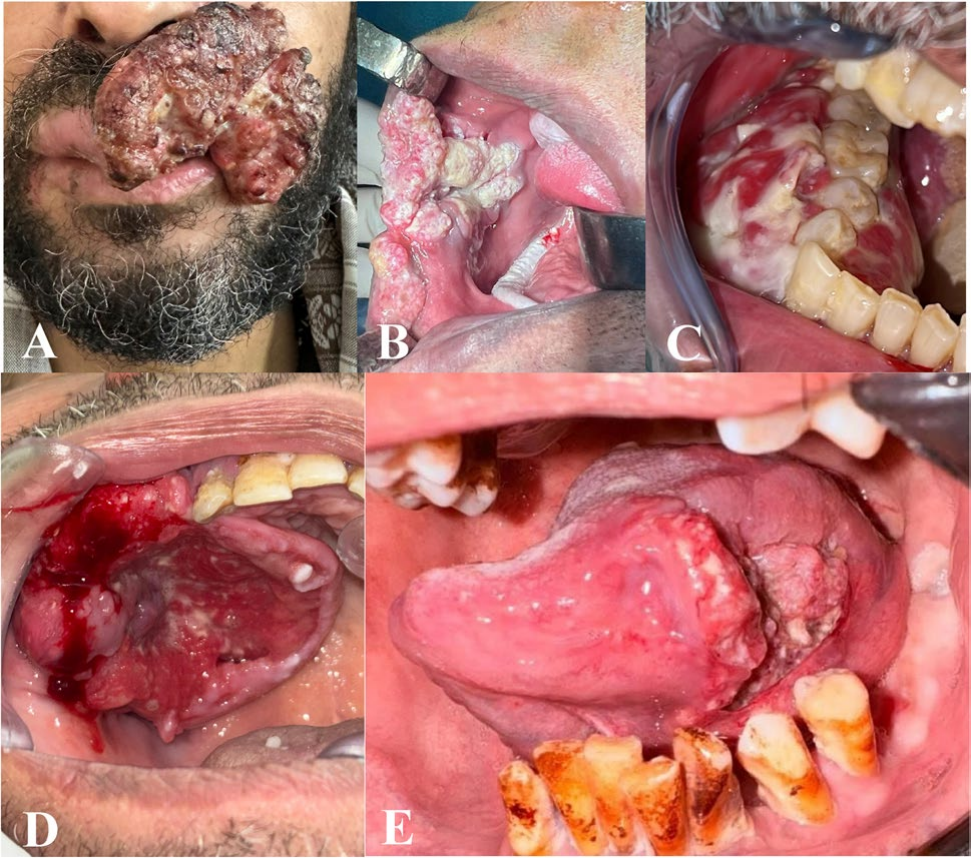
Representative clinical presentations of Oral Squamous Cell Carcinoma (OSCC) at different anatomical sites, illustrating the diverse clinical appearance and extent of disease. **A** Large exophytic fungating ulceroproliferative mass involving more than two-thirds of the upper lip and philtrum, extending superiorly to the base of the nose, representing locally advanced upper lip OSCC. **B** Exophytic erythematous tumor of the right buccal mucosa extending from the commissural area to the parotid papilla posteriorly. **C** Expansile mixed red and white lesion involving the right mandibular alveolus from the canine to the retromolar area. **D** Fungating ulcerative palatal tumor crossing the midline and invading the upper buccal mucosa, demonstrating locally extensive disease. **E** Endophytic ulcerative lesion involving the left side of the tongue and crossing the midline, consistent with advanced tongue OSCC

Oral squamous cell carcinoma (OSCC) is the most common oral malignancy, representing up to 80–90% of all malignant neoplasms of the oral cavity [6]. Histopathologically, OSCC is classified into three grades ranging from well-differentiated (Grade I) to poorly differentiated tumors (Grade III) [7]. Curative surgical resection and reconstruction remain the most common treatment modalities aiming to preserve both function and aesthetics of the head and neck region [2]. Representative clinical presentations of OSCC in different anatomical sites are illustrated in Fig. 1.

OSCC is most common in men in the 6th to 8th decades of life and is rare in patients younger than 40 years [8]. Smoking, betel quid, and tobacco chewing habits are the factors which cause high incidence in vast population of South East Asia [9]. 

Despite recent breakthroughs in treatment modalities, OSCC still has poor prognosis, due to local aggressiveness and metastasis, where recurrence arises in 30% of cases [10]. Local and regional recurrences are the main cause of OSCC-related mortality, where the 5-year survival drops from 92% in recurrence-free patients to 30% in patients with recurrence [10, 11].

The 8th edition of the American Joint Committee on Cancer (AJCC) introduced major modifications in OSCC staging through incorporation of depth of invasion (DOI) and extranodal extension (ENE), aiming to improve prognostic stratification and prediction of survival outcomes. Several studies have demonstrated that AJCC 8th edition provides more accurate assessment of tumor aggressiveness compared to traditional TNM staging. However, validation of these prognostic parameters remains limited in Middle Eastern populations, particularly in Egypt [12]. 

While the new staging criteria have improved prognostic stratification, their correlation with traditional histopathological predictors like tumor grade, LVI, and PNI remains inconsistent across different clinical settings. Furthermore, previous studies focused on single prognostic factors rather than providing a comprehensive analysis of the relationship between demographic factors, staging, and survival outcomes in a localized condition. This highlights the need for clinical studies that validate these prognostic factors in our specific patient population to improve management protocols.

This study aims to assess demographic, clinical, and histopathological characteristics of OSCC patients and to evaluate associations between tumor features (grade, stage) and survival outcomes.

## Materials and methods

This retrospective observational study included all histopathologically confirmed OSCC patients treated at the Department of Craniomaxillofacial and Plastic Surgery, Alexandria University, between January 2018 and December 2022.

Ethical approval was obtained from Committee of Alexandria University Faculty of Dentistry (IRB No. 00010556 – IORG 0008839 with Ethical No: 1104-06/2025). All procedures performed in the study were conducted in accordance with the ethical standards given in the 1964 Declaration of Helsinki, as revised in 2013 and other ethical guidelines adopted by the Research Ethics Committee of Alexandria University Faculty of Dentistry.

Inclusion criteria involving patients diagnosed with histopathologically confirmed primary OSCC, underwent curative surgical resection and availability of complete pretreatment workup (imaging, endoscopy reports) with 5 years follow-up data [13, 14]. Patients with previous head and neck malignancy, synchronous primaries, palliative treatment intent, insufficient tissue for analysis (< 1 cm² tumor area) and neoadjuvant therapy all were excluded from the study [13, 15, 16]. Tumors with an area less than 1 cm² were excluded to ensure adequate tissue availability for reliable histopathological assessment. Patients who received neoadjuvant therapy were excluded because treatment-related histopathological changes could affect evaluation of the studied pathological variables.

### Data collection [17, 18]

Medical records, histopathological reports, and radiographic data were retrospectively reviewed for all OSCC patients who were admitted to the department of craniomaxillofacial & plastic surgery at Alexandria university in the interval between January 2018 till December 2022 and extracted the necessary data, mainly from clinical examination and reports of histopathology & radiographs.

The data included were:

A. Clinical Data (Electronic Medical Records).

1- Demographic (gender, age).

2- Clinical history included medical conditions (e.g., diabetes mellitus, hepatitis, and hypertension), smoking status, addiction history, alcohol consumption, and family history of cancer. Smoking status was obtained from the medical records and categorized as smoker or non-smoker at the time of hospital admission. Alcohol consumption was recorded from the medical records and categorized as present or absent at the time of hospital admission. Addiction history was defined as the documented use of illicit or prohibited substances and was categorized as present or absent. Detailed smoking-related variables, including smoking duration, smoking intensity, pack-years, and former smoking status, were not consistently available in the medical records and therefore were not included in the analysis.

3- Anatomical site of the lesion (tongue, buccal mucosa, floor of mouth, mandible, palate and lower lip).

4- Follow-up data: Patients were followed for five years to assess survival status (alive/deceased) and recurrence status (yes/no). Complete follow-up data were available for all included patients, with no loss to follow-up.

B. Histopathological Review:

Histopathological data were extracted from the final postoperative histopathological reports of the resected specimens.

1- Tumor differentiation: categorized as well, moderately or poorly differentiated [7]. 

2- Margin status was recorded from the final pathology reports and classified as clear (≥ 5 mm) or involved/close (< 5 mm) [19]. 

3- Depth of invasion (DOI) was recorded as reported by the pathologist, measured microscopically from the basement membrane of the adjacent normal mucosa to the deepest point of tumor invasion, and categorized into < 5 mm, 5–10 mm, and > 10 mm according to the AJCC 8th edition criteria [12].

4- Perineural invasion (PNI) and lymphovascular invasion (LVI): recorded as present or absent.

5- Extranodal extension (ENE) was recorded from the final pathology reports and defined as extension of metastatic tumor cells beyond the lymph node capsule into the surrounding soft tissues; it was categorized as present or absent.

C- Staging parameters.

Tumors were initially staged according to the traditional TNM system and subsequently re-staged according to the 8th edition of the American Joint Committee on Cancer (AJCC) incorporating depth of invasion (DOI) and extranodal extension (ENE) [12]. 

D. Outcome Correlation [20].

Recurrence and survival rates were correlated with clinical data, histopathological findings and staging parameters.

E-Histopathological Risk Assessment.

Patients were stratified according to adverse histopathological features. Tumors with depth of invasion (DOI) ≤ 10 mm were considered low risk, while tumors with DOI > 10 mm were considered high risk. In addition, cases showing combined perineural invasion (PNI) and lymphovascular invasion (LVI) were categorized as having a high histopathological risk pattern.

**Fig. 2:**
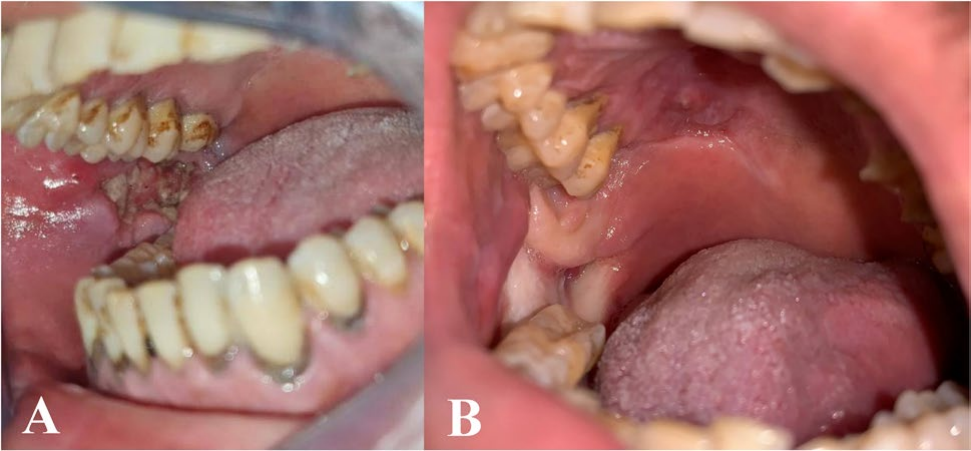
Representative surgically managed case of Oral Squamous Cell Carcinoma (OSCC) demonstrating successful long-term treatment outcome. (**A**) A 63-year-old male presenting with an exophytic ulcerative mass measuring approximately 3 × 5 cm involving the left side of the tongue. (**B**) Five-year postoperative follow-up following tumor resection, ipsilateral neck dissection, reconstruction using a Facial Artery Musculomucosal (FAMM) flap, and adjuvant radiotherapy (33 sessions), demonstrating complete healing, excellent flap integration with the tongue, restoration of functional continuity, and absence of clinical evidence of local recurrence

A representative surgically managed OSCC case with successful postoperative outcome is demonstrated in Fig. 2.

### Statistical analysis

Data management and statistical analysis were performed using Statistical Package for Social Sciences (SPSS) software, version 25.0 (IBM Corp., Armonk, NY, USA). Descriptive statistics were used to summarize the demographic, clinical, staging, and histopathological characteristics of the studied patients.

Categorical variables, including gender, tumor site, TNM stage, AJCC stage, histopathological differentiation, margin status, perineural invasion (PNI), lymphovascular invasion (LVI), extranodal extension (ENE), and combined histopathological risk pattern, were presented as frequencies and percentages. Continuous variables, including age and follow-up duration, were expressed as mean ± standard deviation (SD) and range. Data normality was assessed using the Kolmogorov–Smirnov and Shapiro–Wilk tests. Normally distributed continuous variables were compared between groups using Student’s t-test. Associations between categorical variables and the primary outcomes (survival status and recurrence status) were analyzed using the Chi-square test (χ²) or Fisher’s exact test when expected cell counts were less than 5. All statistical tests were two-sided, and a p-value ≤ 0.05 was considered statistically significant.

Multivariable regression analysis was performed to evaluate the association between selected clinicopathological variables and the studied outcomes (survival status and recurrence status). Regression coefficients (B), 95% confidence intervals (CIs), and corresponding p-values were reported.

No time-to-event survival analyses (Kaplan–Meier curves, log-rank test, or Cox proportional hazards regression) were performed. Therefore, the reported findings represent associations between clinicopathological variables and survival status or recurrence status rather than identification of independent prognostic factors.

## Results

A total of 150 patients diagnosed with Oral Squamous Cell Carcinoma were included in the study. Age ranged from 30 to 83 years, with a mean age of 55.8 ± 11.6 years. Females represented a higher proportion (52.7%) compared to males (47.3%). Tongue was the commonest affected site (46%), followed by the mandible (21.3%), buccal mucosa (11.3%), and floor of mouth (10%), while the palate showed the lowest frequency (4.7%). (Table 1)

**Table 1:** Demographic and clinical characteristics of the studied patients (*n* = 150)

	Number	Percent
Age (years)		
Range	30.00–83.00	
Mean ± SD	55.8 ± 11.6	
Sex		
Female	79	52.7
Male	71	47.3
Site of Lesion		
Buccal Mucosa	17	11.3
Floor of Mouth	15	10.0
Lower Lip	10	6.7
Mandible	32	21.3
Palate	7	4.7
Tongue	69	46.0

*SD* Standard deviation

Diabetes mellitus and hypertension were the most common recorded comorbidities (16.7% each), while hepatitis was reported in 4.0% of patients. Positive family history of cancer was reported in 48.7% of patients, whereas 51.3% reported no family history of cancer (Table 2).

**Table 2:** Distribution of patients according to medical history (*n* = 150)

	Number	Percent
Medical history		
Hepatitis	6	4.0
Diabetes Mellitus	25	16.7
Hypertension	25	16.7
Family History of Cancer		
No	77	51.3
Yes	73	48.7

Regarding tumor staging according to the traditional TNM classification, stage III was the most frequent (39.3%), followed by stage II (26%), stage IV (21.3%) and stage I (13.3%) (Table 3).

**Table 3:** Distribution of patients according to traditional TNM Stage (*n* = 150)

TNM Stage	Number	Percent
I	20	13.3
II	39	26.0
III	59	39.3
IV	32	21.3

Regarding patients’ habits, most patients reported no history of alcohol consumption (93.3%) or addiction (86.7%), while alcohol consumption and addiction were reported in 6.7% and 13.3% of patients, respectively. Smoking was reported in 53.3% of patients, whereas 46.7% were non-smokers (Table 4).

**Table 4:** Distribution of patients according to Habits (*n* = 150)

Habits	Number	Percent
Alcohol		
No	140	93.3
Yes	10	6.7
Addiction		
No	130	86.7
Yes	20	13.3
Smoking		
No	70	46.7
Yes	80	53.3

Histopathological assessment showed that surgical margins were free in 117 patients (78.0%), while involved margins were reported in 33 patients (22.0%). Regarding tumor differentiation, moderately differentiated tumors were the most frequent pattern, observed in 78 patients (52.0%), followed by well-differentiated tumors in 46 patients (30.7%) and poorly differentiated tumors in 26 patients (17.3%). Depth of invasion was > 10 mm in 99 patients (66.0%), while 30 patients (20.0%) had DOI < 5 mm and 21 patients (14.0%) had DOI between 5 and 10 mm. Lymphovascular invasion and perineural invasion were each positive in 100 patients (66.7%) and negative in 50 patients (33.3%). Accordingly, combined LVI and PNI were observed in 100 patients (66.7%), while 50 patients (33.3%) showed no LVI or PNI. Extranodal extension was present in 80 patients (53.3%) and absent in 70 patients (46.7%) (Table 5).

**Table 5:** Histopathological characteristics of the studied patients (*n* = 150)

	Number	Percent
Margin status		
Free	117	78.0
Involved	33	22.0
Tumor differentiation		
Well	46	30.7
Moderately	78	52.0
Poorly	26	17.3
Depth of invasion (DOI in mm)		
< 5 mm	30	20.0
5–10 mm	21	14.0
> 10 mm	99	66.0
Lymphovascular Invasion (LVI)		
Positive	100	66.7
Negative	50	33.3
Perineural Invasion (PNI)		
Positive	100	66.7
Negative	50	33.3
Histopathological risk pattern		
No LVI or PNI	50	33.3
Both LVI and PNI	100	66.7
Extranodal extension (ENE)		
No	70	46.7
Yes	80	53.3

*DOI* Depth of invasion, *LVI *Lymphovascular invasion, *PNI *Perineural invasion, *ENE *Extranodal extension, *mm *Millimeter

According to the American Joint Committee on Cancer 8th edition staging system, stage IVb was the most frequent stage, reported in 79 patients (52.7%), followed by stage IVa in 31 patients (20.7%). Stages I and III were each observed in 16 patients (10.7%), while stage II represented the lowest frequency (5.3%). Regarding clinical outcomes, recurrence was reported in 81 patients (54.0%), while 69 patients (46.0%) showed no recurrence. Similarly, 81 patients (54.0%) were deceased during the follow-up period, whereas 69 patients (46.0%) remained alive (Table 6).

**Table 6:** Distribution of patients according to AJCC staging, recurrence, and survival status(*n* = 150)

	Number	Percent
Staging (AJCC)		
Stage I	16	10.7
Stage II	8	5.3
Stage III	16	10.7
Stage IV a	31	20.7
Stage IV b	79	52.7
Recurrence		
No	69	46.0
Yes	81	54.0
Survival status		
Alive	69	46.0
Dead	81	54.0

*AJCC* American Joint Committee on Cancer

No statistically significant association was detected between survival status and demographic variables. Females represented 49.3% of the alive group and 55.6% of the deceased group, while males represented 50.7% and 44.4% of alive and deceased patients, respectively.

The mean age was 56.5 ± 10.7 years among alive patients and 55.1 ± 12.3 years among deceased patients, with no statistically significant difference between both groups (*p* > 0.05) (Table 7).

**Table 7:** Association between survival status and demographic characteristics

	Survival status				X^2^	*P* value
	Alive **“***n* = 69”		Dead **“***n* = 81”			
Gender	No	%	No	%		
Female	34	49.3	45	55.6	0.590	0.273
Male	35	50.7	36	44.4		
Age						
Range	36.0–83.0		30.0–81.0		0.529	0.468
Mean	56.5		55.1			
SD	10.7		12.3			

*SD* Standard deviation

χ² was used for categorical variables, whereas Student’s t-test was used for continuous variables

No statistically significant association was observed between survival status and tumor site or traditional TNM staging (*p* > 0.05). The tongue was the most frequent site among both alive (43.5%) and deceased patients (48.1%), followed by the mandible, which represented 17.4% and 24.7% of alive and deceased patients, respectively. Regarding traditional TNM staging, stage III represented the most frequent stage in both alive (37.7%) and deceased patients (40.7%), followed by stage II (26.1% and 25.9%, respectively), while stage I showed the lowest frequency among deceased patients (11.1%) (Table 8).

**Table 8:** Association between survival status and clinicopathological characteristics

	**Survival status**				**X** ^**2**^	**P value**
	**Alive** **“***n* = 69”		**Dead** **“***n* = 81”			
	**No**	**%**	**No**	**%**		
Site of Lesion					4.588	0.468
Buccal Mucosa	9	13.0	8	9.9		
Floor of Mouth	9	13.0	6	7.4		
Lower Lip	4	5.8	6	7.4		
Mandible	12	17.4	20	24.7		
Palate	5	7.2	2	2.5		
Tongue	30	43.5	39	48.1		
TNM Stage					0.806	0.848
I	11	15.9	9	11.1		
II	18	26.1	21	25.9		
III	26	37.7	33	40.7		
IV	14	20.3	18	22.2		

*SD* Standard deviation

χ² was used for categorical variables, whereas Student’s t-test was used for continuous variables

No statistically significant association was detected between survival status and hepatitis, diabetes mellitus, hypertension, alcohol consumption, or addiction history (*p* > 0.05). Smoking and positive family history of cancer demonstrated statistically significant associations with mortality (*p* = 0.001). Nearly all deceased patients were smokers (98.8%), while no smokers were reported among alive patients. Similarly, positive family history of cancer was more frequent among deceased patients (82.7%) compared to alive patients (8.7%) (Table 9).

**Table 9:** Association between survival status and medical history, habits, and family history

	Survival status				X^2^	*P* value
	Alive **“***n* = 69”		Dead **“***n* = 81”			
	No	%	No	%		
Hepatitis	2	2.9	4	4.9	0.404	0.419
Diabetes Mellitus	15	21.7	10	12.3	2.367	0.094
Hypertension	15	21.7	10	12.3	2.367	0.094
Alcohol	2	2.9	8	9.9	2.916	0.082
Addiction	12	17.4	8	9.9	1.821	0.134
Smoking	0	0.0	80	98.8	146.03	0.001*
Family History of Cancer	6	8.7	67	82.7	81.717	0.001*

*SD *Standard deviation

χ² was used for categorical variables, whereas Student’s t-test was used for continuous variables

A statistically significant association was observed between survival status and several histopathological parameters (*p* = 0.001). Patients with involved surgical margins showed higher mortality rates compared to patients with free margins. Regarding tumor differentiation, moderately differentiated tumors represented the most frequent pattern among deceased patients (65.4%), followed by poorly differentiated tumors (27.2%), whereas well-differentiated tumors were more common among alive patients (58.0%). Depth of invasion was strongly associated with survival outcome, as tumors with DOI > 10 mm represented 90.1% of deceased cases, while tumors with DOI < 5 mm accounted for only 2.5% of deceased patients. The combined histopathological risk pattern (both LVI and PNI) was significantly associated with mortality, being present in 95.1% of deceased patients, whereas absence of both LVI and PNI was more frequent among alive patients (66.7%). Extranodal extension (ENE) also demonstrated a statistically significant association with survival outcome, being present in 87.7% of deceased patients and absent in 87.0% of alive patients (Table 10).

**Table 10:** Association between survival status and histopathological characteristics

	**Survival status**				**X** ^**2**^	***P*** ** value**
	**Alive** **“***n* = 69”		**Dead** **“***n* = 81”			
	**No**	**%**	**No**	**%**		
Margin status					36.040	0.001*
Free	69	100.0	48	59.3		
Involved	0	0.0	33	40.7		
Tumor differentiation					46.98	0.001*
Well	40	58.0	6	7.4		
Moderately	25	36.2	53	65.4		
Poorly	4	5.8	22	27.2		
Depth of invasion (DOI in mm)					48.051	0.001*
< 5 mm	28	40.6	2	2.5		
5–10 mm	15	21.7	6	7.4		
> 10 mm	26	37.7	73	90.1		
Histopathological risk pattern					63.88	0.001*
No LVI or PNI	46	66.7	4	4.9		
Both LVI and PNI	23	33.3	77	95.1		
Extranodal extension (ENE)					83.33	0.001*
No	60	87.0	10	12.3		
Yes	9	13.0	71	87.7		

*DOI* Depth of invasion, *LVI *Lymphovascular invasion, *PNI *Perineural invasion, *ENE *Extranodal extension, *mm *Millimeter

χ² was used for categorical variables, whereas Student’s t-test was used for continuous variables

A statistically significant association was detected between survival status and American Joint Committee on Cancer staging (*p* = 0.001). Stage IVb represented the highest frequency among deceased patients (85.2%), whereas stage III showed no deceased cases. Among alive patients, stage IVa was the most frequent stage (33.3%), followed by stage III (23.2%) and stage I (20.3%). Early AJCC stages demonstrated better survival outcomes compared to advanced stages, particularly stage IVb (Table 11).

**Table 11:** Association between survival status and AJCC staging

Staging (AJCC)	Survival status				X^2^	*P* value
	Alive		Dead			
	NO	%	NO	%		
Stage I	14	20.3	2	2.5	77.86	0.001*
Stage II	6	8.7	2	2.5		
Stage III	16	23.2	0	0.0		
Stage IV a	23	33.3	8	9.9		
Stage IV b	10	14.5	69	85.2		

*AJCC*  American Joint Committee on Cancer

χ² was used for categorical variables, whereas Student’s t-test was used for continuous variables

No statistically significant association was observed between recurrence and demographic variables (*p* > 0.05). Females represented 49.3% of non-recurrent patients and 55.6% of recurrent patients, while males represented 50.7% and 44.4% of non-recurrent and recurrent patients, respectively. The mean age was 56.5 ± 10.7 years among non-recurrent patients and 55.1 ± 12.3 years among recurrent patients, with no statistically significant difference between both groups (Table 12).

**Table 12:** Association between recurrence status and demographic characteristics

	Recurrence				Test	*P* value
	No		Yes			
Gender	No	%	No	%		
Female	34	49.3	45	55.6	**X**^**2**^ 0.590	0.273
Male	35	50.7	36	44.4		
Age						
Range	36.0–83.0		30.0–81.0		T = 71.34	0.468
Mean	56.5		55.1			
SD	10.7		12.3			

*SD* Standard deviation

χ² was used for categorical variables, whereas Student’s t-test was used for continuous variables

No statistically significant association was detected between recurrence and tumor site or traditional TNM staging (*p* > 0.05). The tongue was the most frequent site among both non-recurrent (43.5%) and recurrent patients (48.1%), followed by the mandible, which represented 17.4% and 24.7% of non-recurrent and recurrent patients, respectively. Regarding traditional TNM staging, stage III represented the most frequent stage among both non-recurrent (37.7%) and recurrent patients (40.7%), followed by stage II (26.1% and 25.9%, respectively) (Table 13).

**Table 13:** Association between recurrence status and tumor site and traditional TNM stage

	Recurrence				X^2^	P value
	No		Yes			
	No	%	No	%		
Site of Lesion					4.588	0.468
Buccal Mucosa	9	13.0	8	9.9		
Floor of Mouth	9	13.0	6	7.4		
Lower Lip	4	5.8	6	7.4		
Mandible	12	17.4	20	24.7		
Palate	5	7.2	2	2.5		
Tongue	30	43.5	39	48.1		
TNM Stage					0.806	0.848
I	11	15.9	9	11.1		
II	18	26.1	21	25.9		
III	26	37.7	33	40.7		
IV	14	20.3	18	22.2		

χ² was used for categorical variables, whereas Student’s t-test was used for continuous variables

No statistically significant association was detected between recurrence and hepatitis, diabetes mellitus, hypertension, alcohol consumption, or addiction history (*p* > 0.05). Smoking and positive family history of cancer demonstrated statistically significant associations with recurrence (*p* = 0.001). Nearly all recurrent patients were smokers (98.8%), while no smokers were reported among non-recurrent patients. Similarly, positive family history of cancer was more frequent among recurrent patients (82.7%) compared to non-recurrent patients (8.7%) (Table 14).

**Table 14:** Association between recurrence status and medical history, habits, and family history

	Recurrence				X^2^	*P* value
	No		Yes			
	No	%	No	%		
Hepatitis	2	2.9	4	4.9	0.404	0.419
Diabetes Mellitus	15	21.7	10	12.3	2.367	0.094
Hypertension	15	21.7	10	12.3	2.367	0.094
Alcohol	2	2.9	8	9.9	2.916	0.082
Addiction	12	17.4	8	9.9	1.821	0.134
Smoking	0	0.0	80	98.8	146.03	0.001*
Family History of Cancer	6	8.7	67	82.7	81.717	0.001*

χ² was used for categorical variables, whereas Student’s t-test was used for continuous variables

A statistically significant association was detected between recurrence and several histopathological parameters (*p* = 0.001). Involved surgical margins were more frequently observed among recurrent patients (40.7%), whereas all non-recurrent patients had free surgical margins. Regarding tumor differentiation, moderately differentiated tumors represented the most frequent pattern among recurrent cases (65.4%), followed by poorly differentiated tumors (27.2%), while well-differentiated tumors showed the lowest recurrence frequency (7.4%). Depth of invasion was significantly associated with recurrence, as tumors with DOI > 10 mm represented 90.1% of recurrent cases, whereas tumors with DOI < 5 mm accounted for only 2.5% of recurrence cases. Positive lymphovascular invasion (LVI) and perineural invasion (PNI) were each significantly associated with recurrence, being present in 95.1% of recurrent cases. Similarly, the combined histopathological risk pattern (both LVI and PNI) showed a significant association with recurrence, accounting for 95.1% of recurrent cases. Extranodal extension (ENE) was also significantly associated with recurrence, being present in 87.7% of recurrent patients, while absent in the majority of non-recurrent patients (87.0%) (Table 15).

**Table 15:** Association between recurrence and histopathological characteristics

	Recurrence				X^2^	*P* value
	No		Yes			
	No	%	No	%		
Margin status					36.040	0.001*
Free	69	100.0	48	59.3		
Involved	0	0.0	33	40.7		
Tumor differentiation					46.98	0.001*
Well	40	58.0	6	7.4		
Moderately	25	36.2	53	65.4		
Poorly	4	5.8	22	27.2		
Depth of invasion (DOI in mm)					48.051	0.001*
< 5 mm	28	40.6	2	2.5		
5–10 mm	15	21.7	6	7.4		
> 10 mm	26	37.7	73	90.1		
Lymphovascular Invasion (LVI)	23	33.3	77	95.1	18.56	0.001*
Perineural Invasion (PNI)	23	33.3	77	95.1	18.56	0.001*
Histopathological risk pattern					63.88	0.001*
No LVI or PNI	46	66.7	4	4.9		
Both LVI and PNI	23	33.3	77	95.1		
Extranodal extension (ENE)					83.33	0.001*
No	60	87.0	10	12.3		
Yes	9	13.0	71	87.7		

*DOI* Depth of invasion, *LVI *Lymphovascular invasion, *PNI *Perineural invasion, *ENE *Extranodal extension, *mm *Millimeter

χ² was used for categorical variables, whereas Student’s t-test was used for continuous variables

A statistically significant association was observed between recurrence status and AJCC staging (*p* = 0.001). Stage IVb represented the highest frequency among recurrent patients (85.2%), whereas no recurrent patients were classified as stage III. Among non-recurrent patients, stage IVa was the most frequent stage (33.3%), followed by stage III (23.2%) and stage I (20.3%). Advanced AJCC stages, particularly stage IVb, were more frequently observed among recurrent patients (Table 16).

**Table 16:** Association between recurrence status and AJCC staging

Staging (AJCC)	Recurrence				X^2^	P value
	No		Yes			
	No	%	No	%		
Stage I	14	20.3	2	2.5	77.86	0.001*
Stage II	6	8.7	2	2.5		
Stage III	16	23.2	0	0.0		
Stage IV a	23	33.3	8	9.9		
Stage IV b	10	14.5	69	85.2		

*AJCC* American Joint Committee on Cancer

χ² was used for categorical variables, whereas Student’s t-test was used for continuous variables

### Multivariable analysis of factors associated with survival and recurrence

Multivariable regression analysis was performed to evaluate the association between the studied clinicopathological variables and the investigated outcomes. For survival status, smoking and extranodal extension (ENE) remained significantly associated with mortality (*p* < 0.05), whereas family history of cancer, margin status, tumor differentiation, depth of invasion, histopathological risk pattern, and AJCC stage did not demonstrate statistically significant associations after adjustment for the other variables included in the model. Similarly, for recurrence status, smoking and extranodal extension (ENE) remained significantly associated with recurrence, while the remaining variables did not demonstrate statistically significant associations after adjustment (Tables 17 and 18).

**Table 17:** Multivariable analysis of factors associated with mortality

* Model summary							
Model	Sum of Squares		df	Mean Square	F		*P* value
Regression	36.346		8	4.543	701.200		0.0001*
Residual	0.914		141	0.006			
Total	37.260		149				

* Regression coefficients							
Model	Unstandardized Coefficients		Standardized Coefficients	95.0% CI		t	*P* value
	B	Standard Error	Beta	Lower	Upper		
(Constant)	0.996	0.024				41.469	0.000
smoking	0.927	0.029	0.928	0.066	18.13	32.432	0.000
Family History of Cancer	0.002	0.020	0.002	0.424	2.34	0.096	0.924
margin status	− 0.015	0.019	− 0.012	0.082	23.0	− 0.768	0.444
tumor differentiation	− 0.001	0.016	− 0.002	0.528	2.084	− 0.093	0.926
Depth of invasion (In mm)	0.003	0.021	0.005	0.554	1.786	0.140	0.889
Hisotpathological risk pattern	0.019	0.011	0.036	0.494	2.679	1.734	0.085
Extranodal extension ENE	0.057	0.024	0.058	0.731	1.357	2.395	0.018
staging (AJCC)	− 0.002	0.012	− 0.006	0.241	1.63	− 0.185	0.854

*Dependent Variable* survival status, *t* t statistic for the regression coefficient, *B* regression coefficient, *C.I *confidence interval

Dependent Variable: survival rate, *F *ANOVA test, *df* degree of freedom’s was significant if ≤0.05

**Table 18:** Multivariable analysis of factors associated with recurrence

* Model summary								
Model	Sum of Squares	df	Mean Square			F		*P* value
Regression	16.830	7	2.404			251.210		0.0001*
Residual	0.880	92	0.010					
Total	17.710	99						

* Regression coefficients								
Model	Unstandardized Coefficients		Standardized Coefficients	95.0% CI			t	*P* value.
	B	Standard Error	Beta	Lower	Upper			
(Constant)	0.040	0.079					0.503	0.616
smoking	0.901	0.039	0.915	17.0	120.8		22.977	0.000
Family History of Cancer	0.002	0.028	0.003	0.921	3.125		0.082	0.935
margin status	− 0.019	0.024	− 0.021	0.830	2.242		− 0.795	0.429
tumor differentiation	− 0.010	0.023	− 0.013	0.678	1.879		− 0.452	0.652
Depth of invasion (In mm)	0.019	0.071	0.022	0.331	16.767		0.268	0.789
Extranodal extension ENE	0.097	0.041	0.107	1.345	4.945		2.366	0.020
staging (AJCC)	− 0.011	0.036	− 0.028	0.187	1.945		− 0.296	0.768

*Dependent Variable* recurrence status, *t*  t statistic for the regression coefficient, *B* regression coefficient, *C.I *confidence interval

Dependent Variable recurrence, *F *ANOVA test, *df* degree of freedom,* p *was significant if ≤ 0.05

## Discussion

Oral Squamous Cell Carcinoma is an aggressive malignancy with prognosis influenced by multiple clinical, histopathological, and systemic factors [2]. The present study analyzed 150 patients to provide a comprehensive overview of the clinicopathological characteristics of OSCC and to identify factors associated with recurrence and survival outcomes.

The demographic data of the studied patients revealed a mean age of approximately 56 years, with most patients being older than 50 years. Female patients represented a slightly higher proportion (52.7%) compared to males (47.3%). These findings are generally consistent with previous epidemiological studies reporting increased incidence of OSCC among older age groups due to cumulative exposure to carcinogenic risk factors [1, 3]. The tongue was the most frequently affected primary site (46.0%), followed by the mandible (21.3%) and buccal mucosa (11.3%), which is in agreement with previous literature identifying the tongue as one of the most common sites for OSCC development [13].

In the present study, traditional TNM staging did not show a statistically significant association with recurrence or survival outcomes, whereas re-staging according to the 8th edition of the American Joint Committee on Cancer demonstrated a significant correlation with both recurrence and mortality. Incorporation of depth of invasion (DOI) and extranodal extension (ENE) resulted in marked stage migration toward advanced stages, particularly stage IVb, and was associated with improved stratification of recurrence and survival outcomes. These findings are consistent with previous studies reporting that AJCC 8th edition provides superior prognostic performance compared to the traditional TNM system through inclusion of DOI and ENE as major prognostic determinants. Similar stage migration toward advanced disease categories has been described following application of AJCC 8th edition criteria, reflecting the significant impact of DOI and ENE on tumor upstaging and outcome prediction [21].

A considerable proportion of patients in the present study presented with advanced disease stages following AJCC re-staging, particularly stage IVa and IVb. This finding may reflect delayed diagnosis and late clinical presentation, which remain common challenges in developing countries. Similar predominance of advanced-stage OSCC has been reported by Aree J et al. [22], whereas Fabio RP et al. [6] demonstrated a more balanced stage distribution, possibly related to differences in early detection programs, healthcare accessibility, and patient awareness across different populations.

Depth of invasion (DOI) demonstrated a strong association with both recurrence and survival outcomes in the present study. Tumors with DOI > 10 mm represented the majority of recurrent and deceased cases, indicating the strong prognostic impact of deeper tumor invasion. These findings support the concept that increasing DOI reflects more aggressive tumor biology, increased potential for cervical lymph node metastasis, and poorer overall prognosis.

Similarly, extranodal extension (ENE) showed a statistically significant association with both recurrence and mortality. Positive ENE was markedly more frequent among recurrent and deceased patients, emphasizing its role as an indicator of aggressive nodal disease and advanced tumor behavior. The present findings further support the growing evidence highlighting the value of DOI and ENE in risk stratification and outcome assessment among OSCC patients [12].

The significant association observed between ENE and adverse outcomes in the present study is consistent with previous reports identifying ENE as a marker of biologically aggressive disease. Recent studies have demonstrated that ENE is closely related to unfavorable histopathological and tumor microenvironment characteristics, including increased tumor budding, immature desmoplastic reaction, low tumor-infiltrating lymphocyte density, and greater depth of invasion, suggesting that ENE reflects an aggressive tumor phenotype rather than isolated nodal involvement. Furthermore, although ENE remains an important adverse pathological feature, some authors have suggested that the extent of ENE may not always independently predict survival outcomes, highlighting the complexity of its prognostic significance [23–25]. 

Advanced AJCC stages were associated with poorer survival and higher recurrence rates, further supporting the role of AJCC 8th edition staging in risk stratification among OSCC patients.

Additionally, histopathological assessment demonstrated high frequencies of lymphovascular invasion (LVI) and perineural invasion (PNI), both were positive in 66.7% of patients. Furthermore, the combined histopathological risk pattern (presence of both LVI and PNI) showed a significant association with recurrence and mortality. These findings indicate aggressive tumor behavior and support the established role of PNI and LVI as adverse prognostic indicators in OSCC. Compared with Gupta et al. [4], the frequencies observed in the present study were relatively higher, which may reflect the advanced disease stages and delayed presentation observed in the studied population.

Regarding surgical margin status, free margins were achieved in 78.0% of patients, while involved margins were reported in 22.0% of cases. Patients with involved surgical margins demonstrated significantly poorer survival and higher recurrence rates compared to patients with free margins. These findings are consistent with Gupta et al. [4], who emphasized that achieving negative surgical margins remains one of the most important determinants of successful oncologic control and improved prognosis in OSCC patients.

Concerning tumor differentiation, moderately differentiated tumors represented the most frequent histopathological grade (52.0%), followed by well-differentiated tumors (30.7%) and poorly differentiated tumors (17.3%). Moreover, higher tumor grades showed significant associations with recurrence and mortality. This partially differs from the findings of Fabio RP et al. [6], who reported well-differentiated tumors as the predominant histological subtype. However, the higher prevalence of moderately differentiated tumors in the present study may reflect the relatively advanced pathological presentation of many included cases. Similarly, Aree J et al. [22] reported predominance of well-to-moderately differentiated tumors in OSCC patients.

Regarding patients’ medical history and habits, diabetes mellitus represented the most common systemic condition among the studied patients. In addition, positive family history of malignancy was significantly more frequent among patients with poorer outcomes. Smoking showed a statistically significant association with both recurrence and mortality in the present study. However, given the retrospective design and the absence of detailed smoking exposure data, this finding should be interpreted as an observed association rather than evidence of a causal relationship.

Regarding systemic comorbidities, no statistically significant association was identified between diabetes mellitus and recurrence or survival outcomes in the present study. Although previous studies have suggested a potential adverse effect of diabetes mellitus on OSCC outcomes [26], this association was not demonstrated in the current cohort. Further studies with larger sample sizes are recommended to better evaluate the potential impact of systemic metabolic disorders on OSCC outcomes.

Over the five-year follow-up period, recurrence was observed in 54.0% of patients, while 46.0% showed no recurrence. Similarly, mortality was reported in 54.0% of patients, whereas 46.0% remained alive during follow-up. These findings may reflect the relatively advanced pathological and AJCC stages observed in a considerable proportion of the studied patients.

Concerning demographic factors, neither age nor gender demonstrated statistically significant associations with recurrence or survival outcomes. These findings are consistent with previous studies suggesting that demographic variables alone may have limited prognostic value compared to tumor-related biological and pathological characteristics in OSCC patients [5].

Similarly, anatomical tumor site and traditional TNM staging showed no statistically significant correlation with recurrence or survival outcomes. This observation further supports the limitations of conventional anatomical staging in predicting prognosis, particularly in advanced OSCC cases. In contrast, AJCC 8th edition staging demonstrated significant prognostic value through incorporation of DOI and ENE, which better reflect tumor aggressiveness and biological behavior [27].

Histopathological findings demonstrated significant associations with both recurrence and survival outcomes in the present study. Involved surgical margins were associated with higher recurrence and mortality rates, emphasizing the importance of achieving adequate oncologic resection margins during surgical management of OSCC. These findings are consistent with previous studies highlighting margin status as a major determinant of local disease control and long-term prognosis [19].

Similarly, tumor differentiation showed significant associations with recurrence and mortality, with moderately and poorly differentiated tumors being more frequently observed among recurrent and deceased patients compared to well-differentiated tumors. These findings support the concept that higher histological grades reflect more aggressive tumor biology and poorer clinical behavior [28].

Furthermore, lymphovascular invasion (LVI), perineural invasion (PNI), and extranodal extension (ENE) demonstrated strong associations with adverse outcomes, reinforcing their role as indicators of aggressive tumor invasion, regional spread, and poorer prognosis. Similar observations were reported by Hsu et al. [29], who emphasized the adverse prognostic impact of LVI and PNI in OSCC patients, particularly in advanced pathological disease.

Collectively, these findings suggest that biological tumor aggressiveness and histopathological risk factors may provide more reliable prognostic information than conventional anatomical staging alone.

Despite the clinical significance of the present findings, several limitations should be acknowledged. The retrospective design of the study may have introduced selection bias and limited control over potential confounding factors. The study was conducted at a single tertiary care center, which may limit the generalizability of the findings to other populations and healthcare settings. In addition, exclusion of cases with limited tissue availability (< 1 cm²) and patients who received neoadjuvant therapy may limit the generalizability of the findings to these patient groups. Also, some clinical and behavioral data, including smoking history, were obtained from medical records and may therefore be subject to documentation bias. Furthermore, detailed smoking-related variables, including smoking duration, intensity, and former smoking status, were not consistently available in the medical records and therefore could not be analyzed.

Another limitation is the absence of detailed time-to-event data, including exact time to recurrence and time to death, which precluded the performance of survival analyses such as Kaplan–Meier estimation and Cox proportional hazards regression. Consequently, formal time-to-event survival modeling and hazard ratio estimation could not be performed. Although multivariable regression analysis was conducted to evaluate the association between clinicopathological variables and the studied outcomes, the findings should be interpreted as associations with survival status and recurrence status rather than definitive evidence of independent prognostic effects.

Future prospective multicenter studies with larger sample sizes and complete longitudinal follow-up data are warranted to enable multivariable survival modeling and further validate these findings. Nevertheless, the study provides valuable data regarding the associations between AJCC 8th edition staging, DOI, ENE, histopathological risk factors, and clinical outcomes in an Egyptian and Middle Eastern OSCC population.

## Conclusion

The present study demonstrated that AJCC 8th edition staging showed stronger associations with recurrence and survival outcomes compared with traditional TNM staging. Incorporation of depth of invasion (DOI) and extranodal extension (ENE) improved stratification of tumor aggressiveness and disease severity. In addition, histopathological risk factors including tumor differentiation, surgical margin status, lymphovascular invasion (LVI), perineural invasion (PNI), and ENE showed significant associations with recurrence and mortality. Smoking and positive family history were also associated with poorer outcomes. These findings highlight the importance of integrating accurate pathological staging, aggressive surgical management, and systemic medical evaluation to improve patient outcomes and long-term survival in OSCC patients.

## Data Availability

The datasets used and/or analyzed during the current study are available from the corresponding author upon reasonable request.

## Abbreviations

OSCC: Oral squamous cell carcinoma
OC: Oral cancer
SCC: Squamous cell carcinoma
DOI: Depth of Invasion
ENE: Extranodal Extension
AJCC: American Joint Committee on Cancer
PNI: Perineural invasion
LVI: Lymphovascular invasion
